# Design and Modeling of Piezoelectric Nanofilm Actuators for Low-Voltage Powered Microrobots

**DOI:** 10.3390/mi17040434

**Published:** 2026-03-31

**Authors:** Jingxian Lin, Ze Chen, Qingkun Liu

**Affiliations:** 1 National Key Laboratory of Advanced Micro and Nano Manufacture Technology, Shanghai Jiao Tong University, Shanghai 200240, China; chinghsien@sjtu.edu.cn (J.L.);; 2 School of Integrated Circuits, Shanghai Jiao Tong University, Shanghai 200240, China; 3 Institute of Medical Robotics, Shanghai Jiao Tong University, Shanghai 200240, China

**Keywords:** microactuator, piezoelectric, nanofilm, low-voltage actuation, microrobotics

## Abstract

Piezoelectric actuators are essential for sub-millimeter robots and reconfigurable microstructures owing to their advantages, including the ability to operate in air and high-speed response. However, the substantial performance degradation observed in piezoelectric actuators with sub-micrometer thickness poses a critical challenge for the design of low-voltage microactuators capable of achieving large bending curvature. Here we develop a coupled analytical–numerical framework for designing multilayer lead zirconate titanate (PZT) nanofilm microactuators under a low voltage constraint (≤5 V). An analytical multilayer beam model is extended to incorporate thickness-dependent material properties and an interfacial dead layer that reduces the effective electric field at small thicknesses. This enables rapid exploration of curvature and the neutral-axis position as functions of the thicknesses of PZT, electrodes, and the dielectric layer. Two- and three-dimensional finite-element simulations provide complementary predictions of neutral-axis location, voltage-dependent curvature response, and eigenmode shapes. The resulting design maps reveal a non-monotonic optimum for PZT thickness in the few-hundred-nanometer range to maximize the curvature change at low voltages and identify ultrathin top electrodes as a key design lever that enhances bending by reducing parasitic stiffness while shifting the neutral axis favorably. These findings offer quantitative guidelines for designing low-voltage, high-curvature piezoelectric microactuators for microrobotic systems.

## 1. Introduction

Microactuators are fundamental building blocks for microrobotics and microelectromechanical systems (MEMSs), enabling tasks such as precision positioning, micro-manipulation, and high-bandwidth motion generation in compact, batch-fabricated devices. Among available actuation mechanisms, piezoelectric microactuators are particularly attractive because they offer high energy density and fast response while operating efficiently in air and vacuum, and they are compatible with standard microfabrication process for scalable manufacturing [1,2,3].

These advantages have led to widespread adoption of piezoelectric actuators across length scales. In micro-positioning and micro-manipulation platforms, stick-slip nanopositioning stages and compliant microgrippers commonly need tens to hundreds of volts of drive to excite piezo stacks and achieve mechanical amplification [4,5]. In microrobotics, piezoelectric actuatdors have enabled insect-scale flapping-wing micro-aerial vehicles. The bulk PZT bimorph transmissions are typically excited at hundreds of volts (e.g., 200–250 V) to reach useful wingbeat amplitudes [6], while piezoelectric thin-film wing actuators have also been explored at much lower voltages (e.g., 5 $V_{pp}$ at resonance), albeit with limited out-of-plane motion [7].

A central limitation of many piezoelectric actuator implementations is therefore the requirement of high driving voltage. Because the piezoelectric strain scales primarily with the electric field, thick piezoceramic stacks and bimorphs generally demand elevated voltages to reach the operating fields needed for large strain and power density [8,9,10,11]. This high-voltage constraint complicates system integration in untethered microrobots and electronics-compatible MEMSs, where compact, efficient, and low-voltage power delivery is often the dominant bottleneck [8,9,10,11].

To address this constraint, the field has increasingly moved toward piezoelectric thin films with nanometer thickness, which can substantially lower the required voltage by reducing the active-layer thickness while retaining useful transverse coupling [1,2,3,12]. Representative PZT thin-film microactuators have been systematically characterized at the microscale [13], and stacked thin-film PZT actuators have demonstrated tens of micrometers of displacement at 5 V by electrically series-connecting multiple ∼200 nm PZT layers [14]. These demonstrations highlight the promise of thin-film piezoelectrics for low-voltage microactuation.

However, when the piezoelectric layer thickness is shrunk down to the sub-micrometer regime, achieving large out-of-plane deformation becomes challenging. In reported measurements, key electromechanical parameters (e.g., transverse piezoelectric and elastic coefficients) can exhibit strong thickness dependence in sub-micrometer thick PZT films due to processing- and size-related effects, and the effective coupling often does not simply scale inversely with the thickness of PZT film [15,16]. At the device level, the neutral-axis position and composite bending stiffness become highly sensitive to the thicknesses of passive layers (electrodes, oxides, and adhesion layers), so accurate multilayer modeling and design strategies are required to avoid stiffness-dominated stacks with small curvature response [17,18,19]. Despite extensive work on piezoelectric beam modeling, systematic design and validation frameworks that jointly capture thickness-dependent material properties and multilayer neutral-axis engineering remain limited, especially for sub-micrometer PZT microactuators.

In this work, we address this gap by developing an analytical–numerical design framework for piezoelectric nanofilm microactuators optimized for low-voltage operation. We combine a multilayer beam model with finite-element analysis to predict curvature-voltage response, neutral-axis evolution, and resonance trends under realistic fabrication constraints. The resulting framework provides practical design maps for maximizing bending curvature per applied voltage in PZT nanofilm microactuators and supports reproducible design and implementation in standard MEMS processes.

## 2. Theoretical Model for Multilayer Piezoelectric Microactuators

In this section, we establish a coupled analytical–numerical framework to predict the low-voltage bending response of multilayer PZT nanofilm actuators. The analytical model provides closed-form curvature expressions and design strategies. The nonlinear FEM was employed to qualitatively assess large-signal behavior without any data-driven parameter fitting or calibration [17,18,19,20].

### 2.1. The Multimorph Structure of Piezoelectric Microactuators

**Figure 1:**
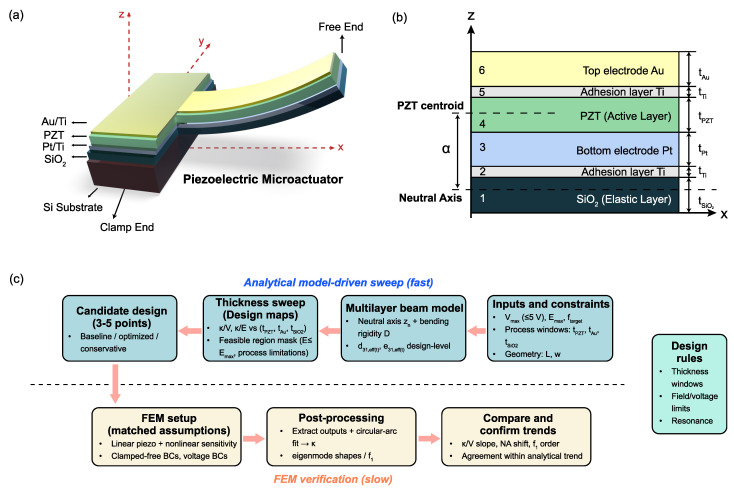
Multilayer unimorph PZT cantilever stack and model variables used in this study. (**a**) Layer stack (Au/Ti top electrode-PZT-Pt/Ti bottom electrode-SiO_2_ support. The Si substrate is removed after release). (**b**) Cross-sectional geometry showing layer thicknesses $t_{i}$, the neutral-axis position $z_{n}$, and the lever arm α from the neutral axis to the PZT centroid. (**c**) Model-guided design workflow for selecting layer thicknesses and predicting curvature (verified by FEM).

The devices studied in this work are piezoelectric cantilever beams, fixed at one end and free at the other (Figure 1a). From top to bottom, each cantilever consists of an Au/Ti top electrode, a sol–gel PZT thin film, a Pt/Ti bottom electrode, a SiO_2_ layer releasing from a Si substrate. The mechanical behavior of the free segment is governed by the Au/Ti-PZT-Pt/Ti-SiO_2_ stack (Figure 1b).

From a processing standpoint, the sol–gel PZT thickness is practically limited to 100 nm–2 μm to enable repeated spin-coating and crystallization without excessive residual stress, cracking, or delamination [12]. PZT crystallization typically involves high-temperature annealing (often around or above ∼600 °C) in an oxidizing ambient [12], which strongly constrains the bottom-electrode choice. Pt is therefore retained for its chemical inertness and thermal stability and for serving as a widely used growth template in thin-film PZT stacks [1,21,22]. Consistent with these process constraints, the Pt/Ti bottom electrode thickness is fixed at 100/20 nm in the present flow to ensure robust adhesion and a continuous, anneal-stable Pt layer. Pt film morphology/stability and adhesion–layer interactions during annealing are known to depend on stress, thickness, and interdiffusion phenomena [23]. As a result, the main geometric design freedom is concentrated in the PZT thickness, the Au/Ti top-electrode thickness, and, within certain bounds, the SiO_2_ thickness.

In contrast, the top electrode can be deposited after PZT crystallization and patterning, enabling broader material choices and more extensive mechanical optimization. Here, an ultrathin Au electrode with a Ti adhesion layer (Au/Ti) is adopted to minimize parasitic bending stiffness while maintaining convenient routing and probing/bonding. Ti is included because Au adheres poorly to oxide surfaces [24,25].

Figure 1c sketches the overall model-guided design flow from material and stack selection to curvature prediction and FEM-based validation.

All geometric and mechanical parameters used in the analytical multimorph model and in the finite-element simulations are summarized in Table 1. The same layer thicknesses and elastic properties are used consistently in both approaches.

With the multilayer stack and geometric variables defined, we next derive a compact curvature expression for a piezoelectric microactuator under low-voltage driving conditions.

### 2.2. Analysis of Bending Curvature Based on a Multilayer Euler–Bernoulli Beam Model

#### 2.2.1. Curvature Expression and Term Definitions

To guide the design of the piezoelectric microactuator, we adopted a multilayer Euler–Bernoulli beam formulation for the Au/Ti-PZT-Pt/Ti-SiO_2_ stack. Euler–Bernoulli kinematics assumes that the deflection is small, that plane sections remain plane and normal to the neutral axis, and that transverse shear deformation is negligible. Under the above assumptions, the bending curvature is related to the internal bending moment through the composite bending rigidity of the multilayer section [17,18,19].

When an electric field *E* is applied across the PZT thickness $t_{\mathrm{PZT}}$, the piezoelectric layer develops an in-plane actuation stress that can be expressed using an effective transverse piezoelectric stress coefficient $e_{31}\left(t_{\mathrm{PZT}}\right)$ for a clamped thin film. This stress produces a resultant axial force in the PZT layer and therefore an internal bending moment about the neutral axis. Denoting the neutral-axis coordinate by $z_{n}$ and the centroid coordinate of the PZT layer by $z_{c,\mathrm{PZT}}$, the mechanical lever arm is defined as [17,19,28,29]

(1) $$\alpha =z_{c,\mathrm{PZT}}-z_{n}.$$

**Figure 2:**
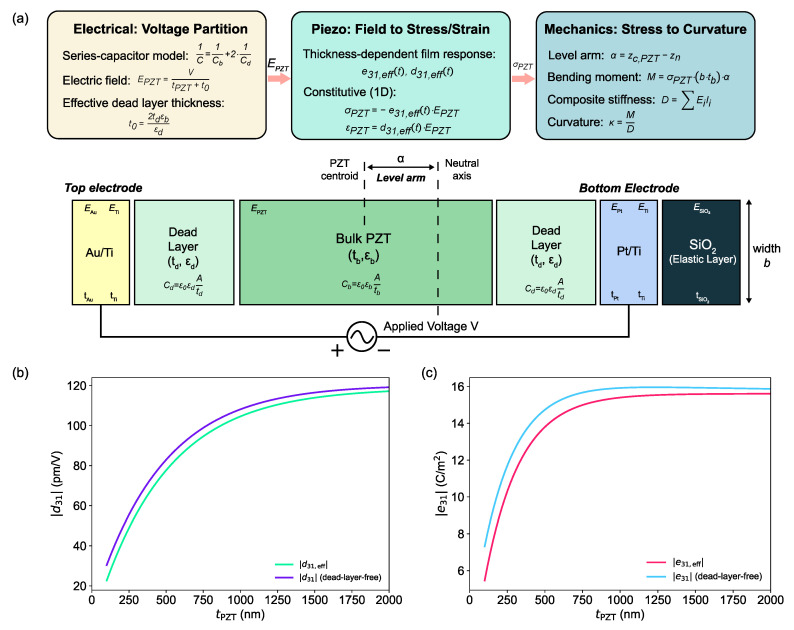
Model hierarchy from intrinsic film coefficients to device-level effective parameters. (**a**) Electro-mechanical block diagram for a voltage-driven thin-film piezoelectric microactuator: the applied voltage *V* is first converted to the internal field $E_{\mathrm{PZT}}$ through an interface/dead-layer (series-capacitor) partition and then mapped to stress/strain via $d_{31}$ or $e_{31}$, producing a bending moment *M* and curvature κ through multilayer beam mechanics. (**b**) Thickness dependence of $\left|d_{31}\left(t\right)\right|$: comparison between the dead-layer-free intrinsic coefficient (reflecting thickness-dependent piezoelectric degradation such as clamping/microstructure effects but excluding voltage-partition effects) and the device-level effective coefficient $\left|d_{31,\mathrm{eff}}\left(t\right)\right|$ after incorporating interface/dead-layer voltage partition. (**c**) Thickness dependence of $\left|e_{31}\left(t\right)\right|$: comparison between the dead-layer-free intrinsic coefficient and the corresponding device-level effective coefficient $\left|e_{31,\mathrm{eff}}\left(t\right)\right|$ after incorporating interface/dead-layer voltage partition.

The coefficient-to-device model hierarchy used in the present framework is summarized in Figure 2.

For the unimorph configuration considered here, the induced bending moment can be written in the compact form:

(2) $$M\left(E\right)=e_{31}\left(t_{\mathrm{PZT}}\right)\;\cdot \;E\;\cdot \;A_{\mathrm{PZT}}\;\cdot \;\alpha ,$$

where $A_{\mathrm{PZT}}$ is the cross-sectional area of the PZT layer (for a beam with a uniform-width *w*, $A_{\mathrm{PZT}}=w\times t_{\mathrm{PZT}}$ with beam width *b*). The resulting curvature follows directly from κ=M/D:

(3) $$\kappa \left(E\right)=\frac{e_{31}\left(t_{\mathrm{PZT}}\right)\;\cdot \;A_{\mathrm{PZT}}\;\cdot \;\alpha }{D}E,$$

where *D* is the composite bending rigidity of the multilayer stack evaluated about the neutral axis (Section 2.2.3).

This expression makes explicit that maximizing curvature at a given electric field requires: (i) increasing the lever arm α (i.e., shifting the neutral axis away from the PZT centroid via electrode/dielectric engineering) and (ii) reducing the composite rigidity *D* while maintaining a sufficiently large effective coupling $e_{31,\mathrm{eff}}$. In the nanofilm regime, $e_{31}$ (and the corresponding $d_{31}$) becomes thickness-dependent due to clamping and microstructural scaling, which must be accounted for to avoid overestimating curvature [14,15,16,17,19,21,30].

In the following section, we parameterize the effective transverse piezoelectric coefficients to capture the reported degradation of transverse coupling in sub-micrometer PZT films.

#### 2.2.2. Thickness-Dependent Piezoelectric Coefficients

Maximizing curvature by thinning the PZT and reducing the composite stiffness must be balanced against the degradation of the effective piezoelectric coefficients in very thin films. Such degradation is commonly attributed to a combination of substrate clamping, microstructural scaling, and interfacial passive layers, all of which become increasingly important in the sub-micrometer regime [15,16,28,31,32,33]. As summarized in Figure 2b,c, the intrinsic thickness dependence of the transverse coefficients should be distinguished from the additional reduction arising from interface/dead-layer voltage partition at the device level.

To capture this behavior at the design stage without introducing microstructure-specific assumptions, we parameterize the thickness dependence of the effective transverse coefficients using a compact saturation form [15,16,31]:

(4) $$e_{31}\left(t_{\mathrm{PZT}}\right)\approx e_{31}^{\infty }\left[1-\exp\left(-\frac{t_{\mathrm{PZT}}}{\lambda_{e}}\right)\right]\;,$$

(5) $$d_{31}\left(t_{\mathrm{PZT}}\right)\approx d_{31}^{\infty }\left[1-\exp\left(-\frac{t_{\mathrm{PZT}}}{\lambda_{d}}\right)\right]\;,$$

where $e_{31}^{\infty }$ and $d_{31}^{\infty }$ represent bulk-like saturation values for sufficiently thick films, and $\lambda_{e}$, $\lambda_{d}$ are characteristic thickness scales describing how fast the coefficients approach their bulk-like limits. In this work, these parameters are treated as design-level fitting constants selected to reproduce representative literature values at a reference thickness and to enforce a gradual saturation toward bulk-like behavior at larger thickness of PZT [15,16,31]. The numerical values adopted in the present work are summarized in Table 2.

This phenomenological model reflects two competing trends. On the one hand, thinning the PZT increases the electric field at fixed voltage and reduces the composite stiffness, both of which tend to increase the change in the bending curvature. On the other hand, as the film becomes thinner, the effective piezoelectric coefficients decrease due to stronger lateral clamping, constrained domain-wall motion and reduced grain size, which diminish the electromechanical coupling. As a consequence, the curvature κ exhibits an optimum at a finite PZT thickness, rather than monotonically improving with thinner films. Identifying this optimal range is a central goal of the subsequent design study [15,16,31].

In addition to substrate clamping and grain-size scaling, electrode/PZT interfacial passive layers can introduce a parasitic series capacitance (“dead layer”), which reduces the effective capacitance/permittivity of the stack and, at a fixed applied voltage, lowers the electric field actually sustained by the ferroelectric layer. This effect is commonly represented by a bulk PZT layer (thickness $t_{\mathrm{PZT}}$, permittivity $\varepsilon_{b}$) in series with two low-permittivity interfacial layers (each of thickness $t_{d}$, permittivity $\varepsilon_{d}$), yielding the familiar thickness-dependent effective permittivity [32,33]

(6) $$\frac{1}{\varepsilon_{\mathrm{eff}}\left(t_{\mathrm{PZT}}\right)}=\frac{1}{\varepsilon_{b}}+\frac{2t_{d}}{\varepsilon_{d}}\frac{1}{t_{\mathrm{PZT}}}\;.$$

At a fixed applied voltage *V*, the series capacitance partitions the voltage across the layered stack, so the average electric field sustained by the ferroelectric layer is reduced relative to the ideal estimate $V/t_{\mathrm{PZT}}$. A convenient design form is [32,33]:

(7) $$E_{\mathrm{PZT}}=\frac{V}{t_{\mathrm{PZT}}+t_{0}},t_{0}=\frac{2t_{d}\varepsilon_{b}}{\varepsilon_{d}},\;$$

which implies the dead-layer (voltage-partition) factor

(8) $$f_{d\left(t_{\mathrm{PZT}}\right)}=\frac{E_{\mathrm{PZT}}}{\frac{V}{t_{\mathrm{PZT}}}}=\frac{t_{\mathrm{PZT}}}{t_{\mathrm{PZT}}+t_{0}}.$$

To obtain the compact series-capacitor form in Equations (6)–(8), we adopt a symmetric effective dead-layer approximation, namely, $t_{d,\mathrm{bot}}=t_{d,\mathrm{top}}=t_{d}$ and $\varepsilon_{d,\mathrm{bot}}=\varepsilon_{d,\mathrm{top}}=\varepsilon_{d}$. This assumption is introduced to keep the voltage-partition model analytically compact for design mapping and should be interpreted as an effective averaged-interface description, rather than a claim that the top and bottom interfaces are physically identical.

For film stacks with asymmetric processing histories, a more general asymmetric series-capacitor form may be used,

(9) $$\frac{1}{C}=\frac{t_{\mathrm{PZT}}}{\varepsilon_{0}\varepsilon_{b}A}+\frac{t_{d,\mathrm{bot}}}{\varepsilon_{0}\varepsilon_{d,\mathrm{bot}}A}+\frac{t_{d,\mathrm{top}}}{\varepsilon_{0}\varepsilon_{d,\mathrm{top}}A},$$

which yields an average field in the ferroelectric layer

(10) $$E_{\mathrm{PZT}}=\frac{V}{t_{\mathrm{PZT}}+\varepsilon_{b}\left(\frac{t_{d,\mathrm{bot}}}{\varepsilon_{d,\mathrm{bot}}}+\frac{t_{d,\mathrm{top}}}{\varepsilon_{d,\mathrm{top}}}\right)}$$

In the present work, since interface-specific capacitances are not independently extracted, we retain the symmetric reduction for compact design exploration.

Consequently, when device-level effective transverse coefficients are interpreted on a per-applied-voltage basis, the dead layer directly suppresses the apparent electromechanical coupling. We therefore write [32,33]

(11) $$d_{31}\left(t\right)=d_{31}^{\infty }\;\cdot \;f_{\mathrm{bulk}}\left(t\right)\;\cdot \;f_{d}\left(t\right)$$

And similarly,

(12) $$e_{31}\left(t\right)=e_{31}^{\infty }\;\cdot \;f_{\mathrm{bulk}}\left(t\right)\;\cdot \;f_{d}\left(t\right)$$

Here $f_{\mathrm{bulk}}\left(t\right)$ summarizes bulk thickness-dependent reductions associated with substrate clamping and microstructural scaling, while $f_{d}\left(t\right)$ captures the dead-layer voltage-partition effect. In the present work, we do not attempt to separately identify these contributions. Instead, their combined influence is lumped into a single design-level thickness factor $f\left(t\right)=f_{\mathrm{bulk}}\left(t\right)\;\cdot \;f_{d}\left(t\right)$, which is parameterized using the exponential saturation form (Equations (4) and (5)) adopted above [32,33].

Using the parameter set listed in Table 2, the coupled effects of intrinsic thickness scaling and dead-layer voltage partition are incorporated into the subsequent curvature calculations. Model parameters are selected from prior representative reports to capture a design-level thickness dependence of microstructural and electromechanical properties [12,15,16,28,31,34,35,36].

With the thickness-dependent coupling determined, we next evaluate how passive layers and electrodes reshape the neutral axis and composite rigidity *D*, which ultimately governs curvature amplification.

#### 2.2.3. Evaluation of Neutral-Axis Coordinate and Composite Bending Rigidity

The neutral-axis coordinate $z_{n}$ and composite bending rigidity *D* are evaluated from standard composite-beam (transformed-section) relations, equivalent to applying the parallel-axis theorem to each bonded layer. We define the thickness coordinate *z* along the stack and compute each layer’s centroid position $z_{c,i}$ from the cumulative layer thicknesses (Figure 1b). For a rectangular layer *i* of width $b_{i}$ and thickness $t_{i}$, the cross-sectional area and second moment of area about its own centroid are [17,18,19]

(13) $$A_{i}=b_{i}t_{i},\;I_{i}=\frac{b_{i}t_{i}^{3}}{12}.$$

Assuming perfect bonding and linear elasticity, the neutral-axis coordinate is obtained by weighting each layer centroid with its axial stiffness:

(14) $$z_{n}=\frac{\sum_{i=1}^{m}E_{i}A_{i}z_{c,i}}{\sum_{i=1}^{m}E_{i}A_{i}},$$

and the composite bending rigidity about the neutral axis is

(15) $$D=\sum_{i=1}^{m}E_{i}\left(I_{i}+A_{i}{\left(z_{c,i}-z_{n}\right)}^{2}\right),$$

where $E_{i}$, $A_{i}$, $I_{i}$, and $z_{c,i}$ are the Young’s modulus, cross-sectional area, second moment of area, and centroid position of layer *i*, respectively, and *m* is the total number of layers in the stack.

In some piezoelectric MEMS designs, electrodes and dielectric layers are treated as negligible, and $z_{n}$ is approximated near the substrate centroid. In this work, since the electrode and dielectric thicknesses can be comparable to $t_{\mathrm{PZT}}$, $z_{n}$ and *D* must be evaluated using the full multilayer section, which directly governs the curvature amplification through $\alpha =z_{c,\mathrm{PZT}}-z_{n}$ and κ=M/D. For patterned electrodes or non-uniform widths, $b_{i}$ is taken as the actual layer width in the FEM-matched geometry, and the same expressions apply [17,37].

With the composite neutral-axis location and bending rigidity fully defined for the multilayer stack, we next employed finite-element simulations to verify the analytical model under FEM-matched geometry, material, and boundary conditions and to extract quantities (e.g., κ-V and eigenmodes) that complement the closed-form formulation.

### 2.3. Electromechanical Simulation Using the Finite Element Method

Finite-element simulations were performed in COMSOL Multiphysics (version 6.3) to benchmark the analytical multilayer beam model under matched assumptions and capture 3D and large-signal effects not represented in the linear beam formulation. All models represent a cantilever of width *w* = 60 μm and length *L* = 300 μm, with a baseline layer stack of SiO_2_ (100 nm)/Ti (20 nm)/Pt (100 nm)/PZT (255 nm)/Ti (3 nm)/Au (20 nm), unless a specific layer thickness is swept (Table 3). The mechanical boundary condition is implemented as an ideal clamped-free support. Specifically, a fully rigid fixed constraint is applied on the entire end face at x=0 (the multilayer cross-section), enforcing $u_{x}=u_{y}=u_{z}=0$ on that boundary, while all other external surfaces are traction-free. The electrical boundary conditions prescribe a potential on the top electrode and ground on the bottom electrode, with all other boundaries electrically insulated; the PZT layer as a piezoelectric material; and the SiO_2_ layer treated as a dielectric.

**Figure 3:**
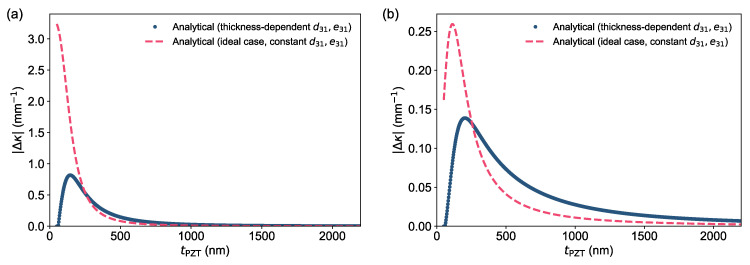
Predicted curvature versus PZT thickness for a cantilever of length 300 μm and SiO_2_ thickness of 100 nm. (**a**) Voltage-limited case (V = 1 V). (**b**) Field-limited case (E = 1 V/μm), representing a breakdown-limited design. Solid curves include thickness-dependent transverse coefficients, and the dashed curve shows an idealized thickness-independent case for comparison.

**Figure 4:**
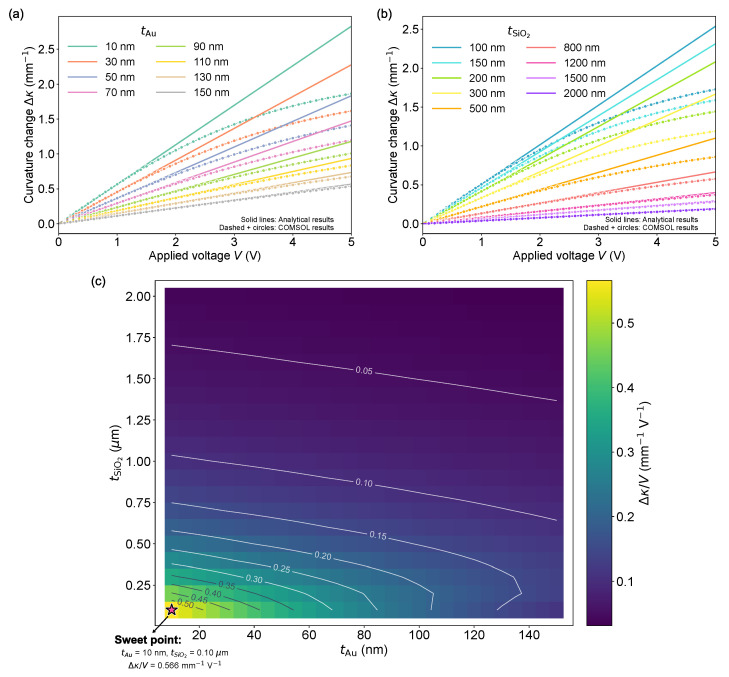
Passive-layer thickness effects on the actuation-induced curvature change. (**a**) Curvature of change Δκ (${\mathrm{mm}}^{-1}$) versus applied voltage for varying Au top-electrode thickness (10–150 nm) at fixed SiO_2_ = 100 nm. (**b**) Curvature of change Δκ (${\mathrm{mm}}^{-1}$) versus applied voltage for varying SiO_2_ thickness (0.1– 2.0 μm) at fixed Au = 20 nm. (**c**) Two-dimensional color map of curvature responsivity, Δκ/V, as a function of Au and SiO_2_ passive-layer thicknesses. The contour lines represent iso-Δκ/V levels, highlighting regions with similar curvature responsivity. The star symbol marks the sweet spot in the scanned design space, and the corresponding optimal thicknesses are indicated alongside the map.

**Figure 5:**
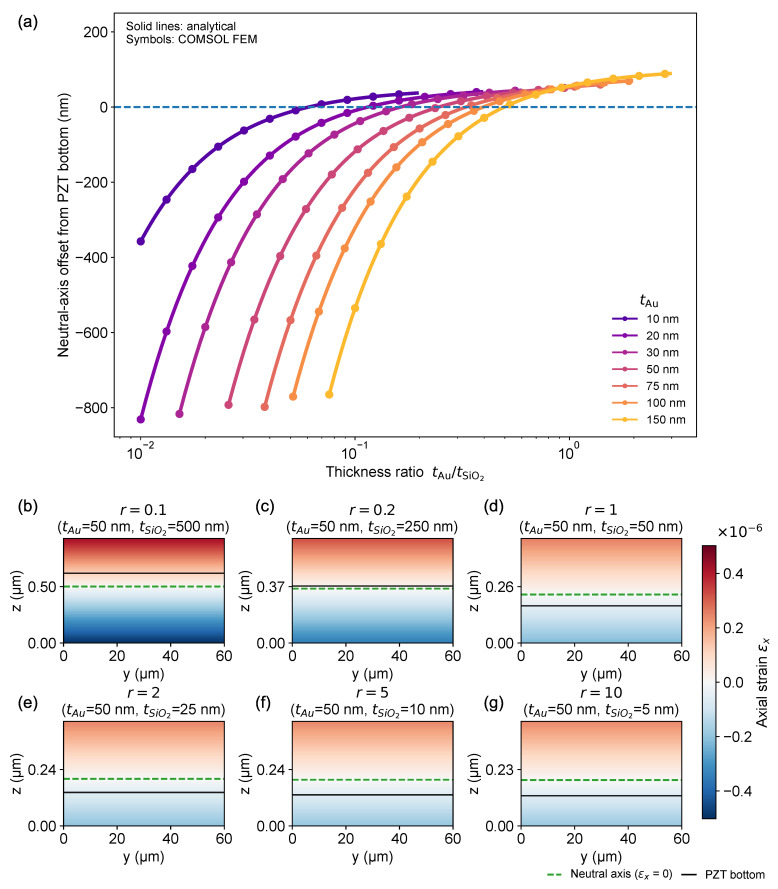
Neutral-axis migration and strain-field interpretation. (**a**) Neutral-axis offset from the PZT bottom interface as a function of the thickness ratio $t_{Au}/t_{SiO_{2}}$ plotted on a logarithmic axis for several fixed Au thicknesses (10–150 nm). Lines: analytical prediction, symbols: COMSOL FEM. (**b**–**g**) COMSOL cross-sectional axial-strain $\varepsilon_{xx}$ maps for a representative case ($t_{Au}$ = 50 nm) in the y-z cross-section (beam width-thickness plane), showing the upward shift of the zero-strain line (neutral axis) and the reduction in internal axial-strain magnitude as $t_{Au}/t_{SiO_{2}}$ increases. Lines: PZT bottom, dashed curves: neutral axis position.

For the parametric studies used in Figure 3, Figure 4 and Figure 5, we employed a computationally efficient 2D longitudinal model (along the x-z plane) with linear piezoelectricity, implemented through the standard coupling of solid mechanics and electrostatics. The PZT constitutive behavior was specified in the strain-charge form, which is directly supported by COMSOL and is consistent with the linear small-signal assumptions used in the analytical model. The mesh was partitioned by layers through the thickness with increased resolution in the PZT layer and discretized along the length to resolve the bending profile (Table 3). Stationary solutions were obtained using a fully coupled solver with a relative tolerance of 1e-3.

**Figure 6:**
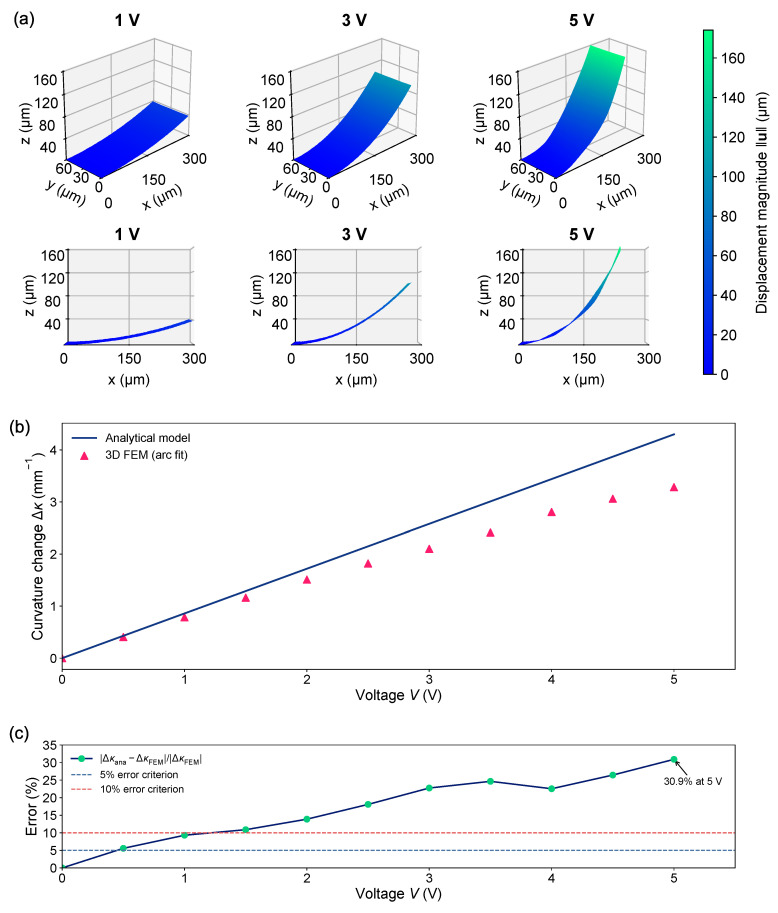
Large-signal electromechanical bending response of a representative cantilever design. (**a**) 3D FEM-predicted deformed shapes from COMSOL stationary simulations with geometric nonlinearity enabled: isometric views (top) and right-side views (bottom) at *V* = 1 V, 3 V, and 5 V. Up to 3 V, the deformed cantilever follows an almost ideal circular-arc shape. At 5 V, a mild inward deflection toward the midline (y=0) becomes visible, indicating weak three-dimensional edge/warping effects superimposed on the primary bending mode. (**b**) Curvature-voltage response (line: analytical multilayer beam model, symbols: 3D FEM), where curvature is extracted by circular-arc fitting of the deformed centerline (y=0) over the electrode-covered region. The FEM matches the analytical linear trend at low voltage but exhibits saturation at higher voltages, yielding smaller curvature than the linear analytical extrapolation. (**c**) Percentage error of the analytical model relative to the 3D FEM reference. The error reaches 30.91% at 5 V. The horizontal gray dashed lines in the lower panel indicate the 5% and 10% error criteria. Based on a 10% error criterion, the analytical model is reliable only up to 1 V, whereas a 5% criterion is not satisfied over the analyzed nonzero voltage range.

**Figure 7:**
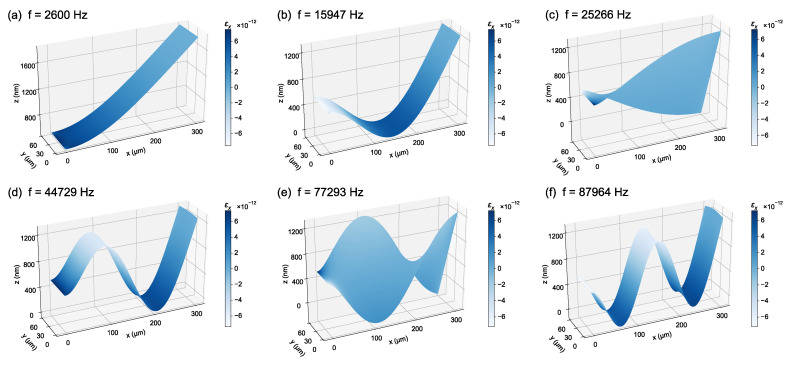
Simulated eigenmodes and longitudinal strain distribution of the cantilever obtained from a 3D eigenfrequency study (linear piezoelectric formulation). The colormap shows the longitudinal strain component $\varepsilon_{x}$ in the multilayer stack. (**a**) Fundamental out-of-plane flexural mode at 2.60 kHz. (**b**) Higher-order out-of-plane flexural mode at 15.95 kHz. (**c**) Torsional (twisting) mode at 25.27 kHz. (**d**) Out-of-plane flexural mode at 44.73 kHz. (**e**) Higher-order torsional mode at 77.29 kHz. (**f**) Out-of-plane flexural mode at 87.96 kHz. For visualization only, the deformed shapes are displayed with a displacement amplification factor of 1.0× and a z-axis visual exaggeration of 400×. Eigenmodes represent relative mode shapes rather than physical vibration amplitudes, which depend on excitation and damping.

To evaluate large-signal deformation and the onset of nonlinear response (Figure 6), a full 3D layered cantilever model was used with geometric nonlinearity enabled so that equilibrium was formulated on the deformed configuration and large-rotation kinematics were captured. The nonlinear stationary problem was solved using Newton’s method with a backtracking strategy and a specified initial damping factor, which improves robustness when the residual is strongly nonlinear. To further enhance convergence, the applied voltage was ramped via an auxiliary continuation parameter ($V=sV_{app}$) using a small set of continuation points (Table 3). Dynamic characteristics (Figure 7) were obtained from a 3D eigenfrequency study using the linearized formulation appropriate for small-amplitude vibration about an equilibrium state.

For curvature-related post-processing, the deformed beam centerline (y=0 in 3D) was exported and fitted to a circular arc to obtain an equivalent radius *R* and curvature κ=1/R. To reduce sensitivity to localized end effects (boundary-layer deformation near the ideal clamp and minor edge effects near the free end), the fit was performed over an interior window that excludes a short margin near both ends. Unless otherwise noted we used x∈ [5 μm, $L_{e}$ − 5 μm], where $L_{e}$ is the electrode length (Table 3).

Having established the analytical–FEM modeling toolkit and the corresponding extraction procedures for κ, the neutral-axis position, and eigenmodes, we next defined the practical fabrication/electrical constraints and curvature-based metrics used to map the feasible design space.

### 2.4. Design Constraints and Curvature Metrics

Because the analytical framework used in this work does not explicitly model residual stress and the corresponding initial pre-curvature, comparisons between designs and between the analytical and FEM results are reported in terms of the actuation-induced curvature change, rather than the absolute curvature

(16) Δκ(V)=κ(V)−κ(0).

Accordingly, curvature responsivity is defined as Δκ/V (${\mathrm{mm}}^{-1}$$V^{-1}$) and was evaluated in the small-signal regime for design mapping (e.g., at 1V in Figure 4c), where both the analytical model and FEM predictions remain approximately linear.

Two practical driving constraints were considered throughout the design study: a voltage-limited condition (constant *V*) and a field-limited condition (constant electric field *E* in PZT), with $E=V/t_{\mathrm{PZT}}$. Under the field-limited constraint, it is also convenient to report Δκ/E as a voltage-independent figure of merit. For representative sub-micrometer PZT stacks, the maximum applied voltage in sweeps is bounded to reflect dielectric-strength considerations. Unless otherwise specified, the results for the baseline $t_{\mathrm{PZT}}$ = 255 nm design are reported up to 5 V as a conservative upper-drive limit used for comparison in Section 3.

## 3. Results

Using the unified multimorph framework and constrained evaluation protocol established in Section 2, we first established the curvature–thickness scaling predicted by this model with thickness-dependent effective coupling and used this relationship to explain the tendency of the curvature change with decreasing the thickness of PZT (Figure 3). The resulting optimal PZT thickness window served as the baseline for subsequent passive-layer optimization, curvature-per-volt design mapping, and low-voltage electromechanical and dynamic evaluations.

### 3.1. Curvature–Thickness Relationship Based on the Multilayer Beam Model

We first examined how the PZT thickness, $t_{\mathrm{PZT}}$, affects the bending curvature while keeping the SiO_2_ and bottom-electrode thicknesses fixed at representative baseline values. For each $t_{\mathrm{PZT}}$, the effective transverse piezoelectric coefficients (e.g., $e_{31}$ and $d_{31}$) were evaluated using the thickness-dependent model introduced in Section 2.2. The multilayer beam formulation was then used to compute the curvature under two practical limiting constraints: (i) voltage-limited operation (constant applied *V*) and (ii) field-limited operation (constant electric field *E* in PZT). In both cases, the neutral-axis position and composite bending rigidity were explicitly evaluated from the full multilayer section [15,16,28,31,32,36].

For voltage-limited operation (Figure 3a, *V* = 1V), the idealized model (dashed curve), which assumes thickness-independent piezoelectric coefficients, predicts that curvature increases monotonically as $t_{\mathrm{PZT}}$ decreases. This trend occurs because thinning the PZT layer reduces the composite stiffness while simultaneously increasing the driving field $E=V/t_{\mathrm{PZT}}$. In contrast, when thickness-dependent coupling is included (solid curve), the curvature becomes non-monotonic: it is strongly suppressed in the ultrathin regime, rises to a pronounced maximum at an intermediate sub-micrometer thickness, and then decreases as the stack becomes stiffness-dominated at larger $t_{\mathrm{PZT}}$. This optimum reflects the competition between electric-field/stiffness leverage and the degradation of effective transverse coupling in very thin PZT.

For field-limited operation (Figure 3b, E = 1 V/μm), when the electric field is held fixed (implemented via $V=Et_{\mathrm{PZT}}$), the curvature change also exhibits a maximum at an intermediate thickness even for the thickness-independent case (dashed curve) because increasing $t_{\mathrm{PZT}}$ rapidly raises the composite bending rigidity and alters neutral-axis leverage. Incorporating thickness-dependent coefficients broadens the curve and can shift the optimum: the curvature change increases from the ultrathin regime as effective coupling recovers, peaks at a moderate $t_{\mathrm{PZT}}$, and then decays more gradually as stiffness dominates.

Overall, the analytical model indicates a practical intermediate $t_{\mathrm{PZT}}$ window that maximizes curvature under both voltage- and field-limited constraints. Importantly, once thickness-dependent coupling is accounted for, the thinnest fabricable PZT does not necessarily yield the largest curvature. FEM validation of the analytical model is provided in Section 3.4 (Figure 6b).

### 3.2. Influence of Passive-Layer Thicknesses Revealed by FEM-Based Neutral-Axis Engineering

We next investigated how passive-layer thicknesses, specifically the Au top electrode and the SiO_2_ dielectric, modulate the bending response through neutral-axis positioning and parasitic stiffness. The PZT thickness was fixed at a representative value (255 nm) within the high-curvature window identified in Section 3.1, while the Pt/Ti bottom electrode was held constant at 100 nm/20 nm to reflect process constraints. The multilayer beam model provided the analytical trends, and 3D COMSOL simulations were used to benchmark the representative one-dimensional cases shown in Figure 4a,b.

In the analytical multilayer beam model (linear piezoelectric constitutive relation + small-deflection beam kinematics), Δκ scales linearly with the applied voltage:

(17) Δκ(V)=S·V,

where the responsivity S=Δκ/V is set by the laminate bending rigidity and the neutral-axis lever arm of the piezoelectric layer. Increasing either $t_{Au}$ or $t_{SiO_{2}}$ increases parasitic stiffness and typically shifts the neutral axis in an unfavorable direction, so both the curvature magnitude and the slope Δκ/V decrease monotonically with thicker passive layers (Figure 4a,b).

Figure 4a shows the curvature change as a function of the driving voltage and $t_{Au}$ at a fixed $t_{SiO_{2}}$ = 100 nm. The FEM results follow the analytical linear Δκ−V relationship up to approximately 1.5 V, confirming that the beam model captures the small-signal electromechanical response and the expected monotonic reduction in slope as $t_{Au}$ increases. Beyond ∼1.5 V, the FEM curves deviate from linearity and exhibit a clear saturating (sub-linear) behavior: the incremental slope d(Δκ)/dV gradually decreases toward zero, and the achieved Δκ becomes lower than the analytical prediction at the same voltage. This behavior is broadly consistent with reported high-field responses of piezoelectric bending actuators, where linear displacement-field scaling holds only under weak driving conditions.

Figure 4b plots the curvature change as a function of the driving voltage and $t_{SiO_{2}}$ at a fixed $t_{Au}$ = 20 nm. The analytical Δκ−V curves remain linear, while increasing $t_{SiO_{2}}$ reduces both Δκ and Δκ/V because SiO_2_ rapidly becomes stiffness-dominant in bending. The FEM again matches the analytical slope at low voltages then transitions into saturation at higher voltages, leading to Δκ values below the analytical prediction.

Figure 4c summarizes the passive-layer design space by mapping the curvature responsivity, Δκ/V, as a function of Au and SiO_2_ passive-layer thicknesses. The pseudocolor map visualizes the magnitude of Δκ/V, while the contour lines denote iso-responsivity levels and highlight regions with similar actuation efficiency. The star marks the sweet spot identified from the scanned design space, and the corresponding optimal thicknesses are indicated alongside the map. Overall, thinner passive layers generally lead to higher curvature responsivity, consistent with the neutral-axis argument above, although the detailed optimum depends on the scanned thickness combinations. This map provides a direct, practical design guideline for selecting passive-layer thicknesses to maximize bending-per-volt within the explored parameter space.

The departure of the FEM results from the linear analytical prediction at V≥1.5 V is consistent with the onset of large-signal nonlinearities that are absent from the simplified beam model. A primary contributor is geometric nonlinearity, where transverse bending induces axial stretching (mid-plane stretching) that effectively stiffens the cantilever and reduces the incremental curvature per added voltage, leading to a saturating Δκ−V trend.

### 3.3. Neutral-Axis Migration and Strain-Field Interpretation

To interpret the passive-layer design trends observed in Section 3.2, we analyzed how the neutral-axis coordinate $z_{n}$ and the composite bending rigidity *D* evolve with the thickness ratio $t_{Au}/t_{SiO_{2}}$ using the same definitions introduced in Section 2.2.3 (Equations (14) and (15)). Here, $t_{\mathrm{PZT}}$ was fixed at a representative value (identical to Section 3.2), and the Pt/Ti bottom electrode stack was kept constant so that the response was governed primarily by the competition between neutral-axis placement relative to the active PZT and parasitic stiffening from passive layers.

Figure 5a compares the neutral-axis offset measured from the PZT bottom interface with the analytical predictions and FEM results, plotted against the thickness ratio $t_{Au}/t_{SiO_{2}}$ on a logarithmic axis. As the ratio increases, the neutral axis shifts upward in a largely monotonic manner: it transitions from below the PZT bottom interface (negative offset) to within the PZT layer (positive offset) and then exhibits a progressively reduced slope, approaching an asymptotic (saturating) trend at large ratios. The sweep over fixed Au thicknesses (10–150 nm) shows a consistent thickness dependence: thinner Au enables the neutral-axis offset to cross zero at a smaller $t_{Au}/t_{SiO_{2}}$, implying that the neutral axis can be brought into the PZT even when SiO_2_ remains relatively thick. In contrast, thicker Au requires a larger ratio to reach the same neutral-axis position, reflecting the stronger influence of the stiff metal electrode in setting the laminate centroid and stiffness. Neutral-axis positioning is a well-established knob in unimorph/bimorph design because it directly controls the strain distribution and bending leverage of the active layer.

The implications of this migration are visualized in Figure 5b–g through COMSOL maps of the longitudinal axial strain, $\varepsilon_{xx}$, plotted on the y-z cross-section (beam width-thickness plane) for a representative electrode thickness ($t_{Au}$ = 50 nm) at several selected ratios. As $t_{Au}/t_{SiO_{2}}$ increases, the zero-$\varepsilon_{xx}$ line, corresponding to the intersection of the neutral axis with the y-z section, clearly shifts upward, passing the PZT bottom interface and moving further into the stack. Concomitantly, the magnitude of $\varepsilon_{xx}$ within the actuator decreases, consistent with a reduced distance between the active layer and the neutral axis. This strain-field view provides a direct physical interpretation of the ratio-dependent neutral-axis trend and confirms that the passive-layer thickness ratio offers a practical, fabrication-relevant knob for positioning the neutral axis relative to the active PZT.

Together, Figure 5 explains why the high-responsivity region in Figure 4c clusters near thin passive layers: designs that limit parasitic stiffness while placing the neutral axis favorably with respect to the PZT thickness maximize bending leverage and therefore enhance Δκ/V in the low-voltage regime.

### 3.4. Electromechanical Response

This section compares the large-signal bending response predicted by the analytical multilayer beam model with 3D finite-element simulations for a representative cantilever design selected from the high-curvature design window established in Section 3.2 and Section 3.3.

#### 3.4.1. Curvature Definition and Extraction

To ensure a consistent comparison, curvature is extracted from the FEM using an equivalent circular-arc fit of the deformed centerline. Specifically, the deformed beam axis along the geometric centerline (y=0) is exported and fitted to a circular arc over the electrode-covered region, yielding an equivalent radius *R* and thus κ=1/R. This fitting-based definition is numerically robust and avoids the noise sensitivity associated with evaluating higher-order spatial derivatives of displacement fields.

#### 3.4.2. Analytical vs. FEM Predictions

Figure 6a shows the 3D deformed shapes predicted by COMSOL stationary simulations with geometric nonlinearity enabled. Isometric views are presented for 1 V, 3 V, and 5 V (top row), together with the corresponding right-side views (bottom row). As the applied voltage increases, the global bending amplitude grows monotonically. Up to 3 V, the deformed cantilever remains well described by a single smooth circular arc over the electrode-covered region, indicating an approximately constant-curvature bending mode. At 5 V, while the out-of-plane bending is still dominant, a mild inward deflection toward the midline (y=0) becomes visible along the beam length, consistent with weak three-dimensional warping/constraint effects. The deformation therefore remains close to a circular-arc centerline, with only a small superposed lateral contraction near the longitudinal edges.

These observations are quantified in Figure 6b through the extracted κ-V response. The analytical multilayer beam model predicts an approximately linear dependence on the applied voltage under linear constitutive assumptions and small-strain beam kinematics. The 3D FEM results follow this near-linear trend at low voltage but begin to deviate from the analytical prediction beyond approximately 1.5 V, with the FEM curvature progressively falling below the linear analytical extrapolation. This sub-linear response is consistent with large-deflection effects captured by the nonlinear 3D formulation but not included in the simplified analytical model, most notably geometric nonlinearity and the associated increase in effective structural stiffness due to axial (mid-plane) stretching as the deformation grows.

To further quantify the validity range of the analytical model, a percentage-error analysis was performed by taking the 3D FEM result as the reference:

(18) $$\mathrm{Error}\left(\%\right)=\frac{\left|\Delta \kappa_{\mathrm{ana}}-\Delta \kappa_{\mathrm{FEM}}\right|}{\left|\Delta \kappa_{\mathrm{FEM}}\right|}\times 100\%.$$

As shown in Figure 6c, the percentage error at 5 V is 30.91%, confirming that the linear analytical model substantially overpredicts the curvature in the high-drive regime. Using a 10% error criterion, the analytical model remains reliable only up to 1 V. A more stringent 5% criterion is not satisfied over the analyzed nonzero voltage range.

Overall, Figure 6 shows that the analytical model is well suited for rapid design screening and trend analysis in the low-voltage regime, where the response remains close to linear. However, as the driving voltage increases and the deformation departs from the idealized constant-curvature assumption, a nonlinear 3D FEM analysis becomes necessary to accurately capture the curvature evolution and the onset of response saturation.

### 3.5. Eigenfrequency Analysis and Modal Shape Characterization

Following the static curvature analyses in Section 3.1, Section 3.2, Section 3.3 and Section 3.4, we characterized the dynamic behavior of the cantilever using a 3D eigenfrequency study in COMSOL. An eigenfrequency analysis determined the structure’s natural frequencies and the corresponding mode shapes by solving the linearized free-vibration eigenvalue problem. Consequently, only the relative deformation pattern of each mode is physically meaningful, while the mode amplitude is arbitrary and depends on subsequent excitation and damping conditions.

Because the eigenmodes represent small-amplitude vibrations about an equilibrium configuration, the PZT layer was modeled here using the linear piezoelectric formulation, which is appropriate for extracting resonance frequencies and mode shapes for resonance-aware design. Unless otherwise specified, the eigenfrequency results were obtained about the nominal equilibrium state (grounded on both electrodes) without an applied dynamic excitation.

Figure 7 summarizes the first six eigenmodes together with the spatial distribution of the longitudinal strain component $\varepsilon_{x}$ in the multilayer stack. Strain-field visualization is particularly relevant to piezoelectric cantilevers because electromechanical transduction depends on the strain developed within the active layer. In cantilever beams, the strain energy density is typically highest near the clamped root, which motivates electrode placement and patterning strategies that emphasize the root region when coupling to dominant flexural modes is desired.

The fundamental resonance occurs at 2.60 kHz (Mode 1) and corresponds to the primary out-of-plane flexural bending mode, with $\varepsilon_{x}$ concentrated near the clamped end. A second out-of-plane flexural mode appears at 15.95 kHz (Mode 2), exhibiting a higher-order deformation pattern. The first torsional mode is observed at 25.27 kHz (Mode 3), where the cross-section shows clear twisting; compared with flexural modes, the associated strain field exhibits stronger lateral variation across the beam width. Higher-order modes are found at 44.73 kHz (Mode 4, out-of-plane), 77.29 kHz (Mode 5, torsional), and 87.96 kHz (Mode 6, out-of-plane), with increasingly complex nodal patterns and alternating tensile/compressive regions along the cantilever.

From an operational perspective, when the device is driven well below the first resonance, its response is dominated by static stiffness and can be treated as quasi-static for low-frequency actuation. In contrast, operation near resonance requires mode-aware drive and layout considerations to avoid inadvertent excitation of torsional or higher-order flexural modes.

## 4. Discussion

### 4.1. Relation to Previous Multilayer Models and Actuator Designs

Classical analytical treatments of piezoelectric cantilever actuators model the structure as an arbitrary laminate and derive closed-form deflection expressions with a constant voltage or constant electric field. These multimorph formulations are powerful for revealing geometric trade-offs—most notably the role of the neutral-axis position and composite bending stiffness—and several works have further refined the stack description by explicitly including bonding layers and electrodes in the elasticity solution [17,18,19].

The present work followed these established multilayer-beam strategies but departed from the traditional assumptions in a way that is critical for a low-voltage, sub-micron piezo-MEMS. First, we treated ultra-thin metal electrodes and the SiO_2_ support layer as mechanically non-negligible constituents: when their thicknesses are comparable to the PZT nanofilm, they can substantially shift the neutral axis and dominate the composite bending stiffness. Second, we introduced a thickness-dependent effective transverse piezoelectric response to capture the widely reported degradation in sub-micrometer films caused by substrate clamping constraints and related size effects. Clamping is widely recognized as a primary reason why conventional ferroelectric/piezoelectric thin films exhibit reduced electromechanical responses when scaled down to sub-micron thicknesses [15,16,17,18,19,28,31,32].

Viewed against the broader literature, the results here clarify why neither very thick films nor ultrathin nanofilms automatically maximize curvature per volt: the optimal Δκ/V emerges only when electrical field leverage, thickness-dependent transverse coupling, and passive-layer stiffness/neutral-axis migration are treated on equal footing [15,16,31].

### 4.2. Design Implications for Low-Voltage, High-Curvature Piezoelectric Microactuators

The coupled analytical/FEM results provide a compact set of design implications for high bending curvature and low-voltage (≤5 V) operation. Most importantly, Δκ/V is maximized in an intermediate sub-micron PZT thickness window rather than at the thinnest fabricable film. Although reducing thickness increases the electric field at a given voltage, this advantage is eventually offset by the degradation of effective transverse coupling captured by the thickness-dependent coefficients introduced in Section 2.2.2 [15,16,31].

Within this optimal thickness window, passive layers become first-order mechanical design variables. The parameter sweeps show that thinning the top electrode is a particularly high-leverage knob: it reduces parasitic bending stiffness and shifts the neutral axis to increase the PZT moment arm, yielding a strong curvature gain per volt. Conversely, excessively thick dielectric/elastic layers rapidly suppress Δκ/V by making the stack passive-dominated and eroding neutral-axis leverage [17,18,19].

Electrical and dynamic constraints should be enforced concurrently with geometric optimization. For very thin PZT, the same single-digit voltages that enable low-power drive can push the internal field toward reliability limits, while overly compliant stacks may compromise bandwidth. The eigenfrequency results indicate that representative designs near the predicted optimum can still maintain kilohertz-scale resonances, supporting fast actuation in microrobotic and reconfigurable microstructure applications [8,9,10,11,38,39].

Cyclic reliability is another important consideration for microrobotic operation, where actuators may experience prolonged repetitive driving. In PZT thin films, degradation is known to depend strongly on both the electrical waveform and the electrode/ferroelectric interface. In particular, fatigue is generally much more severe under full bipolar switching than under unipolar or reduced-switching operation conditions, and defective interfacial dielectric layers can accelerate degradation, whereas cleaner interfaces can markedly improve endurance [40]. More broadly, the long-term reliability of piezoelectric thin films is also influenced by defect chemistry, oxygen-vacancy transport, humidity, self-heating, and mechanically assisted failure pathways, indicating that endurance should be viewed as a coupled electro-mechanical/interface problem rather than a purely geometric one [41]. For the present nanofilm unimorphs, large observable bending does not necessarily imply large local strain in the PZT layer. Under bending conditions, the local strain scales with the distance from the neutral axis, and this distance remains small in an ultrathin multilayer stack. Consistent with this picture, flexible ultrathin PZT films on mica have been reported to retain stable ferroelectric and piezoelectric performance under mechanical bending conditions [42]. Practical endurance in the 100–500 nm thickness regime should therefore be regarded as a coupled outcome of electric-field level, drive waveform, interface quality, and local strain state, rather than thickness alone. Systematic lifetime characterization remains an important subject for future work.

### 4.3. Model Limitations and Outlook

Several simplifying assumptions bound the scope of the present design map. The analytical model relies on small-deflection Euler–Bernoulli kinematics and linear, small-signal piezoelectric constitutive behavior, and the thickness dependence of transverse coupling is introduced phenomenologically rather than identified from experimental data. In addition, the present dead-layer treatment uses an effective symmetric (averaged) interface approximation. Experimentally, the top and bottom PZT/electrode interfaces experience different temperature treatments, leading to differences in interdiffusion, crystallinity, and defect densities. This effect results in different dead-layer thickness and permittivity at both the bottom electrode/PZT and PZT/top electrode interfaces. Stacks with strongly asymmetric interfaces may require separate identification of top- and bottom-interface capacitances and their impact on voltage partition. Furthermore, the present framework does not explicitly model cycle-dependent degradation mechanisms such as polarization fatigue, defect migration, or interface-driven damage accumulation. This omission is intentional because the current study focuses on initial curvature optimization and design mapping, but it also means that long-term endurance must be established experimentally and incorporated in future reliability-aware extensions of the model.

In ultrathin ferroelectrics, strain gradients can generate an additional polarization contribution through flexoelectric coupling between polarization and strain gradient [43]. For bending-dominated cantilevers, a simple estimate can be made by noting that under approximately pure bending conditions the through-thickness strain gradient scales with curvature, $\left|𝜕\varepsilon_{x}/𝜕z\right|\approx \left|\kappa \right|$. Using representative reported flexoelectric coefficients for PZT on the order of μ∼  1 × 10^−6^ C$m^{-1}$ [44], the induced flexoelectric polarization can be estimated as $P_{\mathrm{flexo}}\approx \mu \kappa$. For ${\mathrm{mm}}^{-1}$-level curvatures, this gives $P_{\mathrm{flexo}}\approx$ 1 × 10^−4^ to 1 × 10^−2^ C/m^2^, which is typically a modest fraction of the characteristic polarization scale of PZT [44]. The associated internal field scale $E_{\mathrm{flexo}}\approx P_{\mathrm{flexo}}/\left(\varepsilon_{0}\varepsilon_{r}\right)$ is therefore expected to be modest compared to the applied driving field in the present low-voltage actuation regime. Consequently, flexoelectricity is not expected to dominate the predicted curvature response in our thickness window, but it may introduce a small internal bias or secondary correction under more aggressive downscaling or higher curvature conditions. Incorporating flexoelectric coupling is left for future multiphysics modeling.

We also neglect residual-stress gradients, release-induced warpage, imperfect interfacial bonding, and electrical non-idealities such as electrode resistance, leakage, and explicit interfacial capacitances, all of which can affect absolute curvature and long-term reliability in fabricated devices. Future work will therefore focus on validation of selected stacks within the predicted high-Δκ/V window and on closing the model by incorporating residual stress, nonlinear/large-signal ferroelectric response, and interface/reliability constraints, enabling co-design with low-voltage driver electronics and microrobotic mechanisms [17,18,19,20].

## 5. Conclusions

This paper presented a process-aware, coupled analytical–numerical framework for designing low-voltage multilayer PZT nanofilm unimorph cantilevers with maximized bending curvature. A multilayer Euler–Bernoulli (multimorph) beam model was formulated to explicitly include (i) the composite neutral-axis position and bending rigidity contributions of electrodes and dielectric layers and (ii) thickness-dependent effective transverse piezoelectric coupling relevant to sub-micrometer PZT films. FEM simulations in COMSOL were used to verify the analytical trends with matched assumptions, providing confidence in the model’s suitability for rapid design exploration [17,18,19].

The combined analyses lead to three primary design conclusions for curvature-limited, ≤5 V operation:

(1) PZT thickness exhibits a non-monotonic optimum once nanofilm coupling degradation is included. Therefore, the thinnest feasible film is not necessarily the best choice for curvature per volt.
(2) Top-electrode thinning is a high-leverage design knob because it simultaneously reduces parasitic stiffness and shifts the neutral axis in a way that increases the effective bending moment arm of the PZT layer.
(3) Dielectric/support thickness must be tightly controlled since excessive passive-layer thickness rapidly stiffens the stack and suppresses curvature despite low-voltage drive.

Beyond static curvature, eigenfrequency analysis indicates that representative designs can maintain resonances in the kilohertz range, supporting fast actuation required in microrobotic and reconfigurable microstructure applications. Overall, the presented framework provides physics-based design rules and a validated theoretical baseline for engineering low-voltage, high-curvature PZT nanofilm actuators [8,9,10,11,30,39].

## Figures and Tables

**Table 1:** Material and geometric parameters used in the multilayer beam model and FEM simulations.

Layer	Symbol	Thickness	Young’s Modulus *E* (GPa)	Poisson’s Ratio ν	Notes
Au (top electrode)	$E_{\mathrm{Au}}$	10–150 nm	70–80	0.42–0.44	Typical thin-film Au modulus near bulk (≈78 GPa) [26].
Ti (top adhesion)	$E_{\mathrm{Ti}}$	3 nm	∼110	∼0.34	Bulk Ti values, thin-film effect neglected [26].
PZT	$E_{\mathrm{PZT}}$	100–600 nm	70–120	∼0.30–0.33	PZT thin films often report *E* in this range [1,12,21].
Pt (bottom electrode)	$E_{\mathrm{Pt}}$	100 nm	150–170	∼0.38	Representative modulus for sputtered Pt films [23,26].
Ti (bottom adhesion)	$E_{\mathrm{Ti}}$	20 nm	∼110	∼0.34	Same as top Ti.
SiO_2_	$E_{{\mathrm{SiO}}_{2}}$	100–2000 nm	70–75	0.16–0.20	Thin-film thermal oxide on Si.
Si substrate	$E_{\mathrm{Si}}$	500 μm	130–170	∼0.28	Completely etched for release. Not used in modeling [27].

**Table 2:** Parameters used in the thickness-dependent piezoelectric saturation and dead-layer model.

Parameter	Symbol	Value Used in This Work	Role/Basis
Bulk-like saturation stress coefficient	$e_{31}^{\infty }$	−15.61 C/m	Saturation value in the exponential thickness-dependent $e_{31}\left(t\right)$ model
Bulk-like saturation strain coefficient	$d_{31}^{\infty }$	−118.9 pm/V	Saturation value in the exponential thickness-dependent $d_{31}\left(t\right)$ model
Characteristic thickness scale for $e_{31}$	$\lambda_{e}$	0.232 μm	Characteristic thickness governing the approach of $e_{31}\left(t\right)$ to its bulk-like limit
Characteristic thickness scale for $d_{31}$	$\lambda_{d}$	0.232 μm	In the present parameterization, the same characteristic thickness scale is used for $d_{31}\left(t\right)$
Dead-layer equivalent thickness	$t_{0}$	33.9 nm	Effective thickness used in the voltage-partition factor $f_{d}\left(t\right)=t/\left(t+t_{0}\right)$

**Table 3:** Key COMSOL FEM settings for reproducible simulations.

Item	2D Static Models (Figure 3, Figure 4 and Figure 5)	3D Static Deformation (Figure 6)	3D Eigenfrequency (Figure 7)
Geometry	Cantilever, *w* = 60 μm, *L* = 300 μm (electrode length = 300 μm unless noted)	Same	Same
Baseline layer stack (bottom → top)	SiO_2_ 100 nm/Ti (bot) 20 nm/ Pt 100 nm/PZT 255 nm/ Ti (top) 3 nm/Au 20 nm	Same	Same
Physics	Solid mechanics + electrostatics (linear piezoelectricity), PZT in strain-charge form (matched to analytical $e_{31}$-based parameters)	Layered piezoelectric cantilever, include geometric nonlinearity	Eigenfrequency study with linearized formulation, outputs eigenfrequencies + mode shapes
Mechanical BC	Clamped-free. Fixed constraint at x=0: $u_{x}=u_{y}=u_{z}=0$, on the full end face.	Same	Same
Electrical BC	Top electrode: prescribed V, bottom electrode: ground, SiO_2_: dielectric, PZT: piezoelectric, other boundaries: electrical insulation	Same	Same
Mesh (through thickness)	Layered discretization (elements across thickness): PZT 10; SiO_2_ 5; Pt 4; Ti (bottom) 2; Au 2; Ti (top) 1	Same	Same
Mesh (in-plane)	Along length x: *n* = 100	Along length x: exponential, *n* = 300 “exponent/ratio” = 5. Along width y: *n* = 10	Same 3D mesh settings as Figure 6 (for consistency)
Study/solver	Stationary, fully coupled, relative tolerance 1 × 10^−3^	Stationary, constant Newton, damping factor = 0.7	Eigenfrequency (first 6 modes)
Load ramping/continuation	—	Auxiliary sweep: apply $V=sV_{app}$, sweep points s=(0.5,0.75,1)	—
Curvature post-processing	Exporting deformed centerline and applying circular-arc fitting. κ=1/R	Exporting deformed centerline at y = 0, fit over x ∈ [5 μm, $L_{e}$ − 5 μm] using 200 points, κ=1/R	—

## Data Availability

Data will be made available on request.

## Abbreviations

The following abbreviations are used in this manuscript:

PZT	Lead zirconate titanate (Pb(Zr,Ti)O_3_)
FEM	Finite element method
MEMS	Micro-electro-mechanical system
COMSOL	COMSOL Multiphysics
1D	One-dimensional
2D	Two-dimensional
3D	Three-dimensional
